# A Comprehensive Review of Strategies to Advance Kidney Exchange Programs and Optimize Effectiveness and Outcomes

**DOI:** 10.1016/j.ekir.2026.106539

**Published:** 2026-04-15

**Authors:** Stijn C. van de Laar, Hidde A. de Heus, Annelies E. de Weerd, Matthijs F. Klaassen, Robert J. Porte, Robert C. Minnee, Frank J.M.F. Dor

**Affiliations:** 1 Division of HPB and Transplant Surgery, Department of Surgery, Erasmus MC Transplant Institute, Erasmus University Medical Center, Rotterdam, The Netherlands; 2 Department of Internal Medicine, Nephrology and Transplantation, Erasmus MC Transplant Institute, Erasmus Medical Center, Rotterdam, The Netherlands

**Keywords:** exchange programs, international collaboration, kidney transplantation, living donor

## Abstract

Living donor kidney transplantation (LDKT) offers superior graft survival and cost-effectiveness compared with deceased donor transplantation (DDKT), yet the availability of immunologically compatible living donors limits its reach. Kidney exchange programs (KEPs) can overcome these barriers by matching incompatible pairs through paired exchanges, domino chains, and nonsimultaneous extended altruistic donor (NEAD) chains. This review summarizes the evolution and current landscape of national and international KEPs. We examine operational challenges (cold ischemia time [CIT], logistical coordination, algorithmic fairness, and ethical and regulatory heterogeneity) and explore innovations in matching algorithms, international kidney exchange debates, and machine perfusion technologies. We conclude by proposing strategic priorities to optimize capacity, equity, and outcomes in future kidney exchange efforts.

LDKT offers substantial advantages over DDKT, including improved graft survival and reduced economic costs.^1^^,^^2^ LDKT is preferable because it enables planned procedures and involves shorter CIT. Furthermore, pre-emptive kidney transplantation is more achievable with LDKT, resulting in better graft- and patient-outcomes.^3^, ^4^, ^5^ The superior outcomes of LDKT^6^, ^7^, ^8^ are largely attributed to the careful selection of living donors, who are typically healthier and have fewer comorbidities.^9^^,^^10^ Avoiding complications linked to intensive care stays and brain or circulatory death in deceased donors further enhances graft outcomes.^11^^,^^12^

Despite these benefits, over 50,000 patients in both the United States and the European Union remain on kidney transplant waiting lists, with significant disparities in transplantation rates across different European countries.^13^, ^14^, ^15^ In recent years, the number of transplants has increased across Europe, partly because of the growing use of LDKT.^11^^,^^13^^,^^16^, ^17^, ^18^, ^19^ However, direct LDKT is not always possible because of potential human leukocyte antigen (HLA) or ABO blood group system (ABO) incompatibility between donor and recipient. Incompatible pairs may either undergo transplantation across immunological barriers (ABO-incompatible transplantation and desensitization for HLA-incompatible transplantation) or participate in a KEP, if available.^20^^,^^21^

Transplantation through a KEP offers several advantages over incompatible transplantation, including a less intensive immunosuppressive regimen, generally lower rejection risk, and improved graft survival.^22^^,^^23^ In selected health-economic analyses, KEP has also been associated with lower overall costs and higher quality-adjusted life expectancy compared with incompatible transplantation.^23^ However, not all countries with active LDKT programs have implemented KEPs.^22^, ^23^, ^24^, ^25^ In countries where KEPs exist, these programs have proven effective in expanding transplantation opportunities, offering outcomes comparable to direct LDKT and significantly better than dialysis or DDKT.^17^^,^^24^, ^25^, ^26^, ^27^, ^28^ KEPs are complex, dynamic components of the healthcare system designed to match patients with end- stage renal disease to a compatible donor. This paper elaborates on the status of KEPs and explores opportunities for future improvements and optimization.

### Current Landscape of KEPs

The concept of exchanging incompatible donor-recipient pairs was first introduced by Felyx Rapaport in 1986 in the United States, marking a pivotal moment in the field of LDKT.^29^ This innovative strategy sought to overcome immunological incompatibility between donors and recipients thereby expanding the potential pool of donors. In 1991, this concept was operationalized with the launch of the first KEP in South Korea, demonstrating its clinical feasibility and potential. A key milestone occurred in 2003, when the first national KEP was initiated in the Netherlands.

In the United States, the legal recognition of KEP in 2007 catalyzed further advancements in the field.^30^ This legislative milestone enabled the development of the NEAD chain, which uses bridge donors to maximize the number of transplants originating from a single (unspecified) altruistic donor, creating extended donor-recipient chains.^17^ A major achievement was the successful coordination of a multicenter domino chain including 70 patients in the United States, representing a significant advancement in managing large-scale, complex exchanges. Additionally, the introduction of the voucher program allowed donors to donate a kidney while providing a “voucher” for a recipient to receive a future transplant.^29^

Today, KEPs are active in several countries performing LDKT (Figure 1), including various European countries,^31^ the United States, Canada, Australia, New Zealand, Argentina, and South Korea. These programs vary in scale from large, well-established programs to smaller operational or developing initiatives. The core principle involves matching incompatible donor-recipient pairs with other incompatible pairs to enable mutually beneficial kidney exchanges, thereby expanding the donor pool and increasing the likelihood of successful transplantation for patients with otherwise limited options.

**Figure 1:**
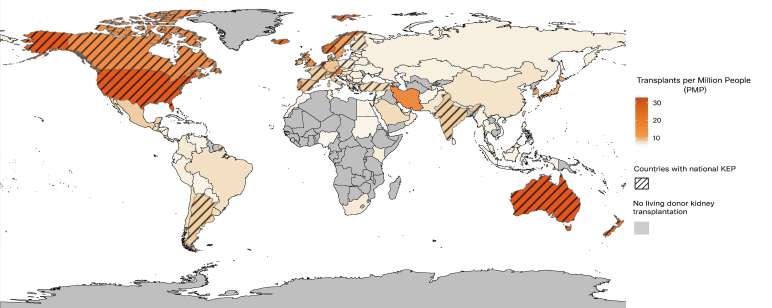
Living donor kidney transplants per country and national Kidney exchange programs (KEP). Countries with a national KEP: Argentina, Australia, Austria, Belgium, Canada, Czech Republic, Denmark, Finland, France, Iceland, India, Italy, Israel, the Netherlands, New Zealand, Norway, Poland, Portugal, Saudi-Arabia, Slovakia, South Korea, Spain, Sweden, Switzerland, Turkey, the United Kingdom, and the United States. A striped country means a national Kidney exchange program is available. Gray means no (data on) living donor kidney transplant is available.

### Exchange Methods in KEPs

KEPs primarily rely on sophisticated matching algorithms to identify compatible donors for incompatible patients. In some cases, compatible pairs may also participate to seek a better HLA match, improved donor-recipient age alignment, because of size mismatch, or for altruistic reasons. Exchanges between centers generally occur through the following 3 approaches^32^:

1. **The donor kidney is transported to the recipient’s transplant center**, resembling the organ distribution and transport logistics used in DDKT (for example in the United Kingdom and the United States). This method is performed in most countries that perform KEP.
2. **The donor travels to the recipient’s center**, allowing both the donor nephrectomy and kidney transplantation to be performed at the same facility, inherently reducing CIT.^21^^,^^31^ India, Saudi Arabia and the Netherlands perform this method, whereas other countries perform both organ shipment and donor travel.
3. **Recipient travels to the donor’s center**, which is considerate to the donor but imposes greater demands on the recipient. Currently only performed in Poland.^32^

In a paired exchange, 2 or more incompatible donor-recipient pairs are arranged in a closed loop where each donor provides a kidney to the next pair’s recipient, and the last donor completes the circle by donating to the first recipient (Figures 2 and 3). Because every donor’s participation is contingent on their intended recipient receiving a kidney, paired exchanges are often, but not universally, performed with simultaneous or near-simultaneous surgeries, to reduce the risk of late withdrawal.

**Figure 2:**
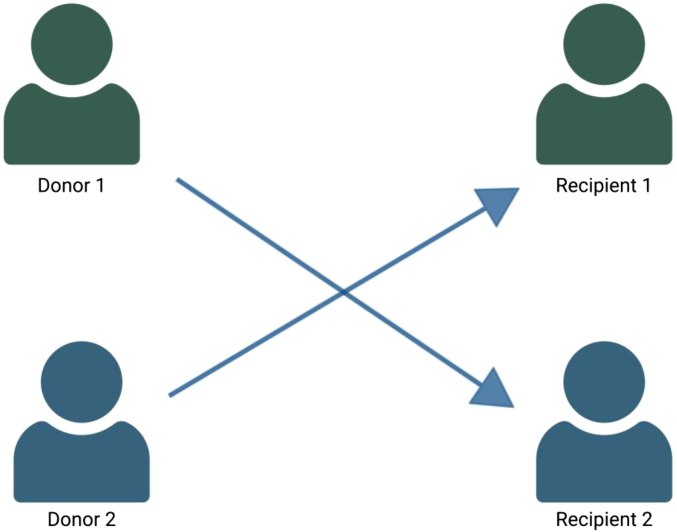
A 2-way kidney exchange where donor 1 and recipient 1 form an incompatible pair, and the arrows indicate donation direction. Created in https://BioRender.com.

**Figure 3:**
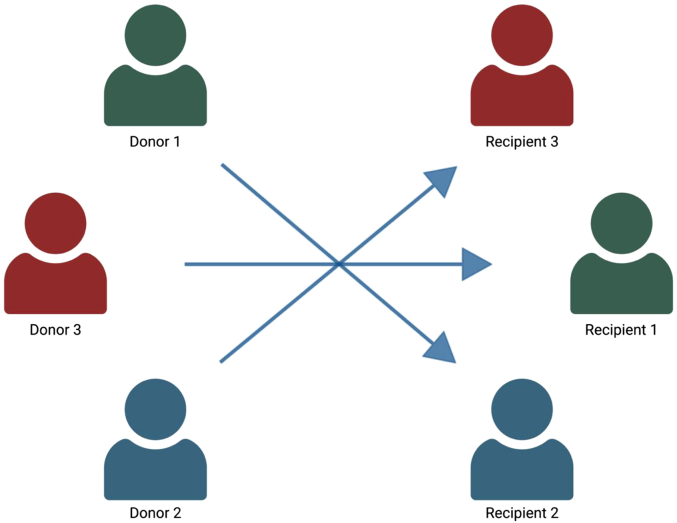
Three-way kidney exchange in which the number correspondents to the original donor-recipient pair, and the arrow indicates the direction of donation. Created in https://BioRender.com.

A domino (open-ended) exchange is initiated by an unspecified donor (UD), also known as nondirected altruistic donor , who, having no designated recipient, donates unconditionally to the first incompatible pair (Figure 4). The recipient’s original donor then donates, propagating the chain to the next pair, and so on until a terminating donor gives to a patient on the deceased-donor waiting list. Because no participant is waiting for a kidney in return, surgeries can be decoupled in time; nonsimultaneous extensions over days or weeks routinely yield chains of an order of magnitude longer than closed cycles, which is then called a NEAD by using a bridge donor (Figure 5).

**Figure 4:**
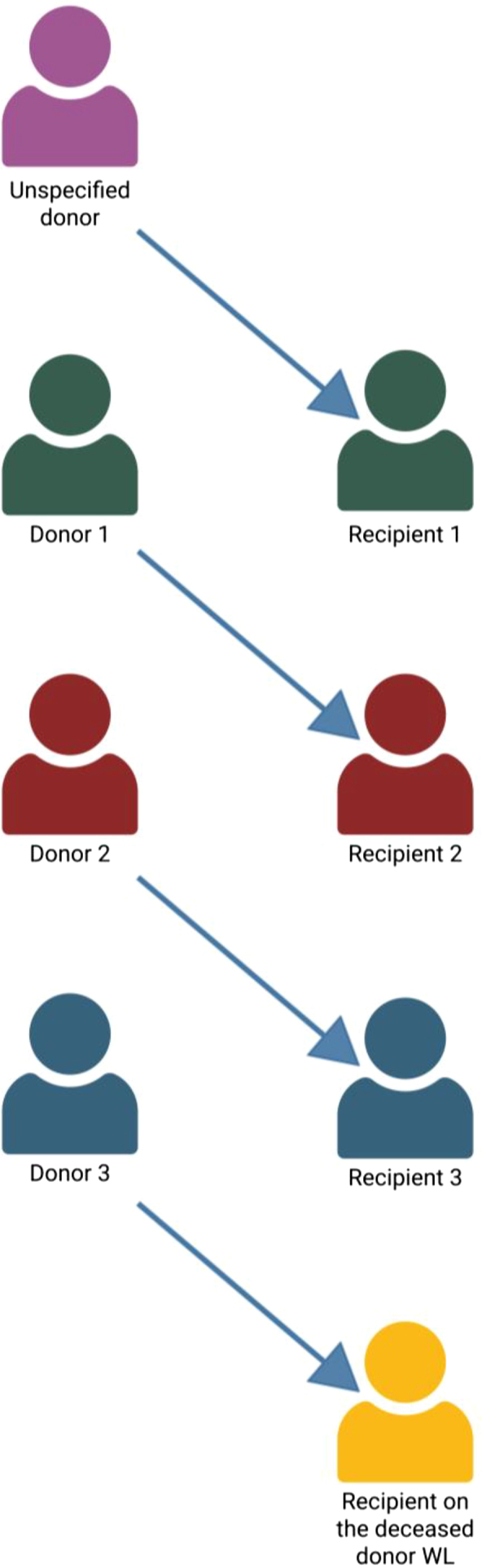
Unspecified (nondirected altruistic) donor-initiated domino chain. Created in https://BioRender.comWL, wait list.

**Figure 5:**
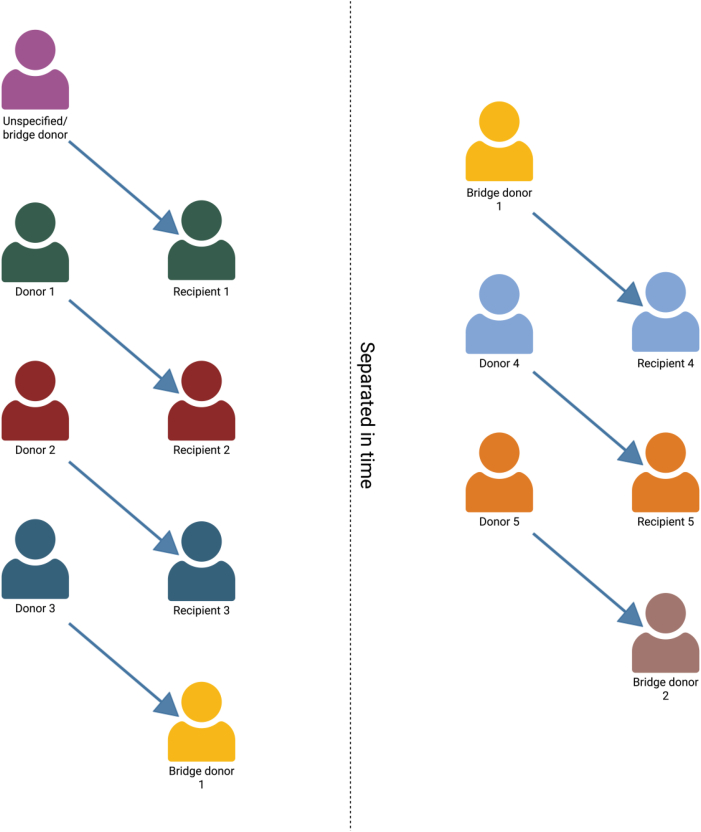
A nonsimultaneous extended unspecified (altruistic) donor chain. The bridge donor can be used to decouple the domino-chains in time. Created in https://BioRender.com.

In some European countries, multidonor registration is allowed which gives donors the option to register more than 1 potential living donor, but only 1 donor ultimately donates if the candidate is matched. Registering multiple donors for 1 recipient is allowed in most European KEPs and not yet in some (Belgium, France, and the Netherlands for example).^33^ This option increases the chances of finding a suitable exchange and, once a recipient is matched, only 1 of the corresponding specified donors proceeds to kidney donation.^34^

Where KEPs exist, additional waiting time after approval of the incompatible donor is common, depending on matching frequency and rates. Matching rates per run are approximately 30% in both the United Kingdom and the Netherlands, compared with around 46% in the United States.^35^^,^^36^

International cooperation in DDKT is already well-established in the form of Eurotransplant and Scandiatransplant, and similar collaborative efforts in LDKT have proven feasible and beneficial, as demonstrated by the Scandiatransplant exchange program.^37^ Despite their success, multiple challenges impede broader international collaboration, including ethical, legal, logistical, medical, and financial barriers.^31^^,^^38^, ^39^, ^40^, ^41^, ^42^, ^43^

Another exchange variant is the deceased donor kidney paired exchange program (DEC-K).^44^^,^^45^ An incompatible pair participating in DEC-K program agrees to the recipient having a high-quality deceased donor kidney (taking a high allocation priority) followed by the living donor in the pair donating to an anonymous recipient (a patient on the waiting list or a recipient in another incompatible pair participating in the program).

A specific operational variant of this approach is referred to as list exchange, in which the benefit to the incompatible pair is realized through prioritized access to the deceased-donor waiting list following donation of the incompatible living donor to a patient on the waiting list.^46^ List exchange is particularly suited for recipients who are difficult to match within conventional paired-exchange cycles and has been formally incorporated into the French kidney exchange program.^47^

From a clinical perspective, participation in DEC-K may be advantageous for selected transplant candidates compared with conventional KEP. Recipients who are highly sensitized, have rare blood groups, or unfavorable HLA profiles may experience prolonged waiting times and low match probabilities within KEPs, even in large programs. For these patients, prioritized access to the deceased-donor waiting list through DEC-K or list exchange may offer a more predictable and timely transplantation pathway. DEC-K may also benefit older or medically complex recipients for whom reduced time to transplantation outweighs the potential advantages of a living-donor graft. At a system level, DEC-K leverages established deceased-donor allocation infrastructures and may be particularly relevant in small or mid-sized KEPs or in settings where large-scale or international living donor exchange is constrained.

In the United States, several KEPs offer specific advantages to donors.^48^ Donors are granted protections, such as prioritization for transplantation if they would develop end-stage renal disease, including for their relatives. Voucher programs enable donors to donate at a convenient time, granting their designated recipient a priority voucher for a future transplant. This model helps to manage logistical challenges and minimize waiting times.^49^

### Optimizing Exchange Models

KEPs have evolved significantly, driven by technological innovations and strategic practices aimed at improving the efficiency and outcomes of LDKT. Contemporary KEPs address multiple forms of incompatibility, including ABO incompatibility as well as immunological barriers, such as HLA mismatching and donor-specific antibodies.

### Donor-Pool Expansion in KEPs

Since their inception, KEPs have relied on algorithmic matching to identify feasible donor-recipient exchanges. Ongoing advances in matching algorithms, combined with increasing computational power and richer clinical data, have progressively expanded the scope and sophistication of KEPs, enabling more precise matching based on a broad array of clinical and logistical variables, including blood type, HLA typing, age, and donor-recipient logistics.^33^ To enhance the efficacy and outcomes of these programs, several key areas require focused attention, as follows: (i) frequency of matching runs, (ii) enlargement and diversification of the donor-recipient pool, (iii) prioritization for highly sensitized recipients, and (iv) algorithm-driven acceptance of selected ABO- or HLA-incompatible matches in cases with low anti-A/anti-B titers or low-median fluorescence intensity donor-specific antibodies.

Theoretically, if an unlimited number of donors existed, matching outcomes could be fully optimized through the application of well-established models. Kidney exchange is formulated as a barter exchange market, in which each donor-recipient pair contributes a kidney and seeks a compatible kidney in return. The objective is to identify an optimal collection of disjoint cycles and chains subject to medical, logistical, and ethical constraints. These problems are typically solved using integer programming and operations research techniques**,** as first formalized for nationwide kidney exchange by Abraham *et al.*,^50^ and further refined in subsequent methodological work. Within the KEP context, enlarging the pool of donor-recipient pairs generally increases the chance of finding immunologically compatible matches and, in turn, improves clinical outcomes.^51^ However, the relationship is not strictly proportional across all program sizes. A steep improvement only up to a certain pool size is expected, after which gains plateau; this trend is also dependent on how highly sensitized the (potential) recipients are. These findings highlight that gains from pool expansion depend not only on size, but also on program design choices, such as cycle length constraints, chain policies, and matching frequency, as emphasized in recent operational analyses.^52^ Simulation-based analyses suggest diminishing marginal returns of pool expansion beyond a certain scale. Using data from 2 US exchanges, Ashlagi *et al.*^53^ quantified this effect— adding just 1 donor every 1 to 2 days markedly improved match rates and shortened waiting times when annual arrival rates were low, whereas the incremental benefit became marginal once programs exceeded roughly 350 to 700 new pairs per year. These results are informative but context-specific, and their quantitative implications may vary across KEPs. Consequently, the impact of pool expansion is greatest in small or mid-sized KEPs, whereas large programs may see diminishing returns and should instead focus on other optimization levers (e.g., algorithmic prioritization of highly sensitized recipients). Finally, Barkel *et al.*^54^ conclude that not only the number of matching runs and the thickness of the pools, but also the departure of recipient-donor pairs impacts the transplant rate in simulation studies. They advise to match frequently to make sure that easy to match recipient-donor pairs do not leave the pool.

Strategies to expand the donor pool include the following:

1. Transnational KEPs, which will be elaborated on in the next section.
2. Inclusion of compatible pairs. Encouraging compatible donor-recipient pairs to participate in KEPs to optimize HLA or age matching can yield significant benefits.^55^^,^^56^ Modelling indicates that a single compatible pair has a 34 % chance of securing a more favorable donor in a center-based program and 48 % in a national registry of the United States. If all compatible pairs participate, the individual chance falls to 11.7 % and 14.7%, respectively, because more pairs compete for the same subset of younger or better-matched donors.^26^ Even so, their enrolment enlarges the overall donor pool, improving access for recipients with rare HLA types or high sensitization levels. Careful timing remains essential to avoid delaying pre-emptive transplantation, which is independently associated with superior outcomes.^26^ Nicolo *et al.*^56^ suggest that the increase in matches mainly arises from the heterogeneity in the donor pool rather than enlarging of the donor pool, which suggests that large donor pools can also benefit from adding compatible donors.
3. Mobilizing ABO-group O donors. Kidneys from type O donors are universally compatible and can therefore serve as powerful “chain starters” in kidney-exchange cycles for ABO incompatible pairs. Yet, these donors remain under-represented in most KEP registries because they are never blood-type incompatible with their intended donor and only participate for HLA-incompatibility with their recipient. Actively integrating type O donors, either by enrolling compatible pairs that include an O-blood-group donor or by recruiting nondirected altruistic donors with blood group O, would allow more exchanges, unlock transplants for highly sensitized or ABO-incompatible recipients, and help mitigate the structural disadvantage faced by O recipients in DDKT allocation. For equity, O-to-non-O exchanges should be embedded within chains or credit mechanisms that ultimately return a kidney to an O recipient.^26^^,^^55^
4. Inclusion of unspecified (non-directed) altruistic donors. Individuals who volunteer to donate a kidney without a designated recipient can be incorporated into KEPs as unspecified (nondirected altruistic) donors. By contributing a kidney without a reciprocal dependency, these donors expand the donor pool and enable exchange mechanisms that extend beyond simple paired exchanges.^30^^,^^57^ The role of nondirected donors as initiators of chain-based exchange strategies, including NEAD chains, is discussed in the following section.

### Expanding Through Chain-Based and Voucher Systems

Building on the contribution of UDs, chain-based exchange strategies enable a single unspecified donation to facilitate multiple transplants. NEAD chains allow the impact of altruistic donation to be extended over time. In NEAD chains, the donor of the last pair in the chain is recruited as a “bridge donor” who can start a new chain segment of incompatible pairs later. The added value of NEAD chains is contingent on program characteristics but simulation work shows meaningful gains of NEAD chains over domino chains when chain segments of a length of 4 to 6 links are permitted^58^ and when the donor pool is large enough to supply timely downstream matches.^59^ Unlike simultaneous exchanges, NEAD chains do not require all transplants to occur simultaneously, since the altruistic donor kick-starts the chain without creating a reciprocal dependency. Each subsequent donation can be scheduled independently reducing the risk of a collapsed swap and easing logistical constraints. This added flexibility allows the chain to extend over time, thereby increasing the total number of transplants facilitated by one UD.^59^ NEADs are already in use in the United States, however, these systems require large donor pools to maximize their potential. Although several European countries use KEPs, many are limited by smaller donor pools and have yet to implement all available exchange options. Future solutions to these limitations will be discussed in the following sections.

Similarly, voucher donation programs, or time-separated paired exchanges, provide substantial flexibility. This system allows donors to schedule their donation according to personal, professional, or health-related preferences while issuing a “voucher” to their intended recipient for future use.^49^ This innovation not only allows donors to specify their donation to a relative but also expedites access for recipients, especially for those who might otherwise face extended waiting times. By increasing both the number and flexibility of living donors, voucher systems enhance the overall efficiency of KEPs and improve recipient outcomes. The National Kidney Registry reports that the availability of voucher programs promotes a decoupling of donation timing and recipient need, with UDs increasingly transitioning to family voucher donation.^60^

However, KEPs can raise ethical issues that extend beyond standard living donor transplantation because allocation decisions can affect multiple recipients simultaneously and also because matching algorithms embed value judgements regarding prioritization, equity, and transparency. Policy frameworks in Europe emphasize the protection of donor autonomy, stringent assessment, and anonymity to reduce coercion and payback risks and note that many KEPs rely on simultaneous surgeries to minimize the possibility that a recipient fails to receive a transplant once their intended donor has already donated.^61^ These ethical safeguards become more complex in advanced donation models and voucher-based programs. Ethical concerns can arise regarding sustainability, enforceability, and informed consent, particularly when vouchers are issued and administered by private organizations. Although concerns have been raised regarding reliance on private organizations to honor future transplant commitments, existing voucher programs have incorporated contractual safeguards to ensure voucher redemption even in the event of organizational failure, and no systematic ethical breaches have been reported to date.^62^ Nonetheless, sustained regulatory oversight, transparency, and public governance remain essential to maintain trust and ethical integrity as KEPs continue to expand. Nationally coordinated voucher frameworks, such as the single centralized national voucher donation program currently under consideration in Canada, may offer a robust model for aligning long-term institutional accountability with public oversight.^63^ In this context, a single centralized national system may also offer a unique opportunity to systematically evaluate the population-level impact of voucher-based donation and, as argued by Cohen *et al.*,^62^ may be better positioned to realize gains in LDKT than fragmented, privately operated models.

### Current Status of European KEPs and the Need for Integration

European KEPs operate within national programs or through collaborations of varying scale— bilateral or trilateral agreements such as Ireland’s participation in the UK Living Kidney Sharing Scheme, the Iberian consortium of Spain, Portugal and Italy, and the Czech–Austrian KEP,^64^^,^^65^ as well as broader multilateral networks like the Scandiatransplant exchange program, which spans all 5 Nordic countries.^37^

Small KEP pools inherently limit the number of possible donor-recipient combinations. Rather than waiting for sufficient donor-recipient pairs and matches, larger KEP pools can be obtained by merging existing ones. Importantly, operational research suggests that the benefits of larger or merged KEPs depend on harmonizing matching algorithms and operational constraints across participating programs, rather than scale alone.^54^ Merging national KEPs into broader international networks would significantly enhance match opportunities and improve outcomes for patients facing immunological barriers.^66^ Integrating national KEPs into a single international network is hampered by legal heterogeneity. Germany, for example, still bans paired exchange over concerns about trafficking, coercion and donor withdrawal,^67^ but currently a law is designed to allow KEP in Germany.^68^^,^^69^ Integration of KEPs is also hampered by ethical and cultural differences, since societal attitudes toward living donation and exchange vary widely across countries. Disparate healthcare and reimbursement systems further complicate cross-border coordination, as funding models, insurance coverage rules, and tariff structures seldom align, while operational and regulatory misalignment add layers of administrative delay.

In international exchanges, the transportation of kidneys rather than donor travel is generally preferable, especially when long travel distances are involved.^70^ Countries where the donor is currently travelling would need to adapt their systems to a hybrid model, using traveling donors for domestic exchanges and transporting kidneys for international KEPs. Evidence suggests that neither system is inferior in terms of graft outcomes or donor quality of life.^12^^,^^17^^,^^71^

### Algorithms Used to Match Donor Recipient Pairs in KEP

KEPs generally formulate matching as a combinatorial optimization problem on a directed compatibility graph, with the aim of identifying optimal sets of disjoint cycles and chains. This framework is most commonly implemented using integer programming and related operations research techniques, as described in early foundational work and subsequent methodological developments.^50^^,^^58^^,^^72^

Although early implementations focused primarily on maximizing the total number of transplants, more recent approaches increasingly incorporate multiple, and sometimes hierarchical, objectives. These may include prioritization of highly sensitized recipients, waiting time, blood group equity, logistical feasibility, and robustness to exchange failure, with trade-offs between efficiency and equity made explicit in the optimization design.^52^^,^^54^^,^^73^

Importantly, the degree of algorithmic transparency varies considerably between programs. Surveys of operational research approaches note that detailed descriptions of matching algorithms are not consistently published for many large, multicenter exchanges, where implementations may be proprietary or only partially disclosed.^52^^,^^54^ European experience further illustrates that algorithmic matching is often embedded within broader governance structures, in which national legal frameworks, ethical norms, and clinical review committees influence or override algorithmic recommendations, as documented in the European Network for Collaboration on Kidney Exchange Programs working group reports.^33^^,^^34^

Consequently, direct benchmarking of program performance and formal comparison of algorithmic design choices remain challenging. Ongoing methodological research seeks to address scalability and complexity through hybrid and hierarchical optimization frameworks capable of handling large pools and competing objectives, although real-world adoption of these approaches remains heterogeneous across programs.^73^^,^^74^

This algorithmic heterogeneity is also evident in how individual national and multinational KEPs implement matching in practice. According to the European Network for Collaboration on Kidney Exchange Programs working group, European programs differ substantially in permitted exchange structures, optimization objectives, and operational safeguards.^34^ The United Kingdom restricts exchanges to pairwise and 3-way cycles to prioritize logistical feasibility and robustness, whereas the Netherlands allows longer cycles and applies iterative reoptimization following failed crossmatches. Southern European programs, including Spain and Italy, emphasize short cycles and regional feasibility, often combined with ranked solution lists from which clinicians select final matches. Multinational programs such as the Scandinavian Scandiatransplant exchange program and the Czech–Austrian KEP operate merged pools and apply optimization models that explicitly balance transplant volume with prioritization of highly sensitized recipients. Smaller European programs may rely on semiautomated or clinician-guided selection among multiple feasible solutions rather than enforcing a single algorithmically optimal outcome.

In contrast, large US exchanges employ large-scale integer programming formulations capable of handling extensive pools, long altruistic chains, and voucher-based mechanisms. These systems incorporate hierarchical objectives and robustness constraints, but detailed algorithmic specifications are not consistently available in the public domain, limiting independent assessment of optimization criteria and trade-offs. Recent methodological work addresses the computational challenges inherent in these large-scale settings through improved scalability, hierarchical optimization, and hybrid solution strategies.^73^

Algorithmic fairness is also a crucial consideration in transnational KEPs.^75^ Klimentova *et al.*^75^ propose formal fairness models for multiagent KEPs that explicitly balance efficiency against equity, ensuring that no participating country or agent is systematically disadvantaged by repeated matching decisions. As Biro *et al.*^33^ highlight, algorithms must ensure equitable matching opportunities across countries, avoiding scenarios where 1 country disproportionately benefits from the pool. In practice, balanced matching systems should therefore incorporate explicit fairness constraints alongside clinical objectives, while also considering transport distance and immunological outcomes.

### Integrating Novel Matching Models and Data-Driven Approaches

An innovative addition to existing KEP frameworks is the Computerized Integration of Alternative Transplantation program.^76^ Although KEPs broadly aim to improve access for difficult-to-match recipients, the Computerized Integration of Alternative Transplantation program introduces explicit prioritization and expanded matching rules for predefined subgroups, notably selected highly immunized and long-waiting patients. Rather than increasing the size of the donor pool, the Computerized Integration of Alternative Transplantation program increases transplantation opportunities within the existing pool by allowing selected ABO-incompatible and/or low-risk HLA-incompatible matches when immunological risks are considered acceptable. In a pilot implementation, this approach increased transplantation rates among difficult-to-match patients without reducing the total number of transplants performed, illustrating a pragmatic balance between equity and efficiency within kidney exchange design.

The parallel emergence of large, linkable transplant registries has opened the door to applying big-data analytics and artificial intelligence methods to kidney exchange optimization.^77^ Advanced statistical and machine learning models can identify complex patterns in donor-recipient compatibility, predict graft survival, assess rejection risks, and optimize donor selection strategies.^78^ A recent proof-of-concept study by Alowidi *et al.*^79^ evaluated a machine learning–based system for donor-recipient compatibility classification, using routinely applied biological criteria, such as ABO compatibility, HLA matching, donor-specific antibodies, and age differences. In this single-center dataset, gradient-boosting models achieved high accuracy in reproducing predefined, rule-based compatibility categories, and a custom ranking approach performed better than simple similarity-based ranking methods. The accompanying web-based platform (Nephron) serves as a user interface for data entry, visualization, and expert review. Although such approaches illustrate the technical feasibility of applying machine learning to aspects of donor-recipient assessment, their added value over established KEP matching algorithms remains to be demonstrated in real-world exchange programs.

### Enhancing Donor Pools Through International Collaboration

In LDKT, enlarging the donor pool is crucial for improving both immunological and logistical matching. The United Kingdom’s single national KEP, managed by National Health Service Blood and Transplant, accounted for approximately 18% of all LDKTs in 2023, while 48% of registered recipients in the UK Living Kidney Sharing Scheme have been transplanted.^80^ In the United States, multiple independent registries coexist— the National Kidney Registry’s voucher-driven chains predominate, whereas the United Network for Organ Sharing KEP itself represents only about 19% of living-donor transplants.^60^ Continental Europe combines strong national programs (e.g., the Netherlands, the United Kingdom, and Spain) with established multinational consortia such as Scandiatransplant (Nordic countries), the Italy–Portugal–Spain consortium and the Czech Republic–Austria–Israel network, resulting in cross-border collaboration.^81^, ^82^, ^83^ Canada and Australia–New Zealand operate unified national registries that integrate nonsimultaneous domino chains and voucher exchanges, with kidney exchange accounting for a substantial proportion of total LDKT activity.^84^^,^^85^ Besides enlarging the donor pool, international exchanges can result in more biological heterogeneity, through HLA diversity. For example, highly sensitized patients in some regions may be less sensitized to donors in other regions.^52^ European simulation studies also show that joined KEPs of several European countries can improve the long-term performance of both national and international KEPs either by increasing the number of transplants implemented or by decreasing the number of crossmatches.^66^ Work has now begun on KEP-SOFT, an allocation software that is going to be used by multiple national European and International KEPs.^86^

### The Role of a Central Coordinating Body

To address the issues of international KEP, a central (European) governing body for KEPs, modeled on the successful Eurotransplant and Scandiatransplant structures, is recommended. Such a body could oversee a standardized legal framework enabling KEP participation, establish common ethical guidelines promoting equity, transparency, and donor/recipient protection, as well as facilitate intercountry coordination and data sharing while complying with data protection regulations. The use of advanced logistic systems and integrated tracking technologies would further minimize risks associated with extended transport times, while ensuring timely and secure kidney delivery.^87^

EURO-KEP^88^ could assume the role of a pan-European coordinating authority once fully operational. It would first harmonize national legal frameworks and ethical standards, drafting unified protocols for consent, data protection, donor and recipient rights, and equity. It would then maintain a single, high-performance matching platform driven by an optimized algorithm that pools all participating registries and negotiates multilateral reimbursement agreements to ensure fair cost-sharing among health care systems. Although keeping distances short where possible, improved predicted outcomes and options for the highly immunized patients are the benefits from international exchange. On the logistical front, EURO-KEP would manage every step of the organ transport chain, standardizing organ packaging, customs clearance and cross-border shipping; coordinating surgical scheduling; and employing real-time tracking and quality-monitoring technologies. By enforcing uniform standard operating procedures and leveraging advanced logistical platforms, it would minimize cold-ischemia times and other delays inherent in extended transport. In doing so, EURO-KEP would eliminate duplicated efforts, streamline exchanges across jurisdictions and unlock the capacity for (longer) NEAD chains and broader international swaps, thereby significantly expanding transplant opportunities throughout Europe.

Beyond the European context, the concept of Global Kidney Exchange has already been piloted by the US–based Alliance for Paired Donation.^89^ Beginning in 2015, the Alliance for Paired Donation matched financially disadvantaged donor–recipient pairs from low- and middle-income countries with immunologically incompatible pairs in high-income nations, using the cost-savings realized by insurers to fund both sides of each transplant. Dubbed “Global Kidney Exchange,” this model aims to expand access to living donation worldwide but has provoked vigorous ethical debate. Nikzad *et al.*^90^ similarly proposed “Global Kidney Chains,” pairing middle-income and high-income country pairs in long-distance chains financed by healthcare savings, and urged the creation of an international body to monitor procedure, funding escrows, and outcomes. Ambagtsheer *et al.*^40^ reviewed its legal, psychosocial and ethical dimensions, acknowledging its potential to help patients, yet emphasizing the need for rigorous safeguards, independent oversight, and strict adherence to nonpayment principles to avoid exploitation.

### CIT and Transport Considerations

One of the primary logistical challenges in international KEPs is the increase in CIT associated with transporting kidneys across borders, as it is generally unfeasible to ask donors to travel long distances for surgery. Although a meta-analysis suggests that CIT exceeding 4 hours has minimal effect on graft survival,^91^ other studies indicate that prolonged CIT may increase the risk of delayed graft function (DGF) and, in some cases, graft loss in LDKT.^50^, ^51^, ^52^, ^53^, ^54^ In a large US study by Treat *et al.*,^70^ shipped kidneys were associated with higher patient mortality compared with in-center exchanges after 3 years, though no significant differences in graft failure rates were observed. Incremental increases in CIT were not independently associated with graft loss or DGF in adjusted analyses but were associated with patient mortality. This finding should be interpreted with caution. Although residual confounding and center-level or recipient-level differences may contribute, a biologically plausible pathway also exists whereby impaired early graft function contributes to increased mortality.

When synthesizing these findings, including the United Kingdom experience^17^ and the meta-analysis,^91^ these data suggest that extended CIT in LDKT is unlikely to substantially compromise graft survival and mortality, but may have clinically relevant implications for patient outcomes. Accordingly, strategies to mitigate CIT-related risk remain important, particularly in recipients with limited physiological reserve. To further mitigate these risks, hypothermic machine perfusion (HMP) might present an effective strategy, particularly for high-risk donors and/or recipients or when extended transport times are anticipated. HMP not only improves graft viability during prolonged CIT but also offers greater scheduling flexibility for transplant centers, optimizing the timing of surgeries.

Numerous studies aim to identify optimal donor-recipient matches^92^^,^^93^ and predict the likelihood of adverse outcomes such as rejection, graft failure, or DGF.^17^^,^^77^^,^^94^^,^^95^ Despite improvements in matching algorithms and transplant logistics, some recipients inevitably carry higher baseline risk factors, including prolonged dialysis vintage, multiple retransplants, high panel reactive antibodies (PRA), and older donors, which increase the likelihood of suboptimal outcomes.

KEP recipients have been shown to have a higher risk of DGF and rejection following transplantation.^12^ DGF in the setting of LDKT is strongly associated with inferior outcomes, including higher rejection rates^96^ and reduced graft survival.^17^^,^^97^^,^^98^ The hazard ratio for graft failure in cases of DGF after LDKT has been reported at 3.25 compared with immediate graft function.^12^ The prevailing hypothesis is that DGF promotes graft inflammation and fibrosis, thereby accelerating dysfunction and eventual graft loss.^18^^,^^99^

As international collaboration in KEP expands, the inevitable increase in CIT poses additional risk for DGF especially considering the rise in extended criteria living donor kidneys, and, potentially, impaired graft survival. A possible solution to shorten the CIT is proposed by Mesnard *et al*.,^100^ by transporting kidney grafts by drone. The development still is in the preclinical phase and must overcome major challenges, but aerial drones could become a crucial logistical component in the future of organ preservation. Another promosing solution that might mitigate these additional risks is machine perfusion technology. In this section, we review current evidence on machine perfusion in DDKT and explore its potential applications within KEP frameworks.

Three machine perfusion modalities are used in DDKT as follows: HMP, hypothermic oxygenated machine perfusion (HMPO_2_), and normothermic machine perfusion (NMP).^101^ We will discuss the evidence in DDKT in the next section.

HMP involves cooling the kidney to 4 to 10 °C, markedly reducing cellular metabolic demand while preserving adenosine triphosphate and limiting harmful metabolic byproducts.^101^ This temperature range inhibits the generation of reactive oxygen species, reducing ischemia-reperfusion injury and preserving endothelial and cellular membrane integrity.^102^ Clinical studies have shown HMP reduces DGF rates, improves early graft function, and enhances 1-year graft survival in DDKT.^103^, ^104^, ^105^

HMPO_2_ advances this approach by oxygenating the perfusate during hypothermic storage, which further supports mitochondrial adenosine triphosphate production and reduces hypoxic injury.^106^ Oxygenated perfusion diminishes the inflammatory immune response triggered by ischemia-reperfusion injury upon reperfusion, thereby decreasing reactive oxygen species production and tissue damage.^102^ A randomized controlled trial comparing HMP to HMPO_2_ in donation after circulatory death kidneys demonstrated a significant improvement in 1-year graft survival (97% vs. 89%), favoring HMPO_2_.^107^

Normothermic machine perfusion (NMP) maintains the kidney at physiological temperatures (37°C) using oxygenated blood or a substitute, sustaining normal metabolism, and continuous adenosine triphosphate generation. It offers the advantage of real-time functional assessment by measuring urine output, electrolyte balance, and waste removal. NMP may reduce ischemic injury by preserving cell viability and promoting tissue repair.^108^ However, NMP requires continuous expert supervision, and, in case of machine failure, the kidney must be cooled immediately to avoid warm ischemia. Moreover, criteria for kidney viability during NMP are still poorly established and validated.

Among these techniques, HMP and HMPO_2_ appear to be the most feasible and clinically valuable options for integration into KEP. Unlike NMP, which requires intensive oversight, HMP and HMPO_2_ can be applied during transport, making them suitable for the logistical realities of national and international KEP.

Although direct evidence in LDKT is limited, 1 nonrandomized study suggests that HMP reduces DGF risk,^31^ with improvements in creatinine clearance at 1 year, although data on long-term eGFR or graft survival were lacking. In contrast, HMP’s efficacy in DDKT has been consistently demonstrated in multiple studies.^33^^,^^49^

It is yet unclear whether LDKT recipients, especially those transplanted via KEP, and who are at higher risk for developing DGF,^53^^,^^109^ graft failure^26^^,^^55^^,^^76^ or rejection,^37^^,^^77^ will benefit from additional treatment options. However, there are some applications for machine perfusion in KEP that might optimize the donor graft. These could be used if the effect on reduced DGF is shown or to prevent adverse outcomes at all costs. First, to facilitate international KEP; machine perfusion could remove a significant barrier to international KEP in cases with long CIT, such as longer than 10 hours. The ability to safely extend preservation times would enable longer-distance exchanges and expand donor pool accessibility. Next, to protect high-risk grafts, kidneys with higher Living Kidney Donor Profile Index^92^ scores for instance, indicating increased risk for DGF or graft loss, could benefit from additional support provided by HMP or HMPO_2_. Lastly, to optimize logistical flexibility, machine perfusion allows for greater scheduling flexibility, facilitating more efficient surgical planning by preserving graft quality for extended periods compared with static cold storage. This can reduce logistical pressures on transplant centers and enable transplantation at the optimal moment for both donor and recipient.

Further research is needed to determine the specific benefits of machine perfusion in LDKT and KEP recipients and to establish protocols for its standardized application within national and international exchange programs.

### Discussion and Future Recommendations

As demonstrated in this overview KEPs possess substantial potential to increase both the volume and quality of LDKT, ultimately improving long-term patient outcomes. To fully harness these benefits, several strategic directions are recommended.

Firstly, countries with established LDKT infrastructures but without operational KEPs should prioritize the development and implementation of such systems. The integration of a well-designed KEP framework is crucial for expanding the potential donor pool and optimizing match quality, both of which are essential for improving transplant success rates and long-term graft survival.

Secondly, for countries already using KEPs, ongoing refinement and optimization are essential. This can be achieved through several strategies as follows: (i) expanding donor registration efforts and promoting the inclusion of multiple potential donors per recipient, thereby increasing the pool size and improving match opportunities, (ii) incorporating additional donor-recipient pairs, such as advanced donor age or suboptimal HLA compatibility into exchange programs, which broadens the pool and increases access for difficult-to-match patients, (iii) integrating complementary systems, such as UD triggered domino chains and voucher-based programs which can enhance flexibility and further improve long-term outcomes for both donors and recipients, and (iv) including compatible pairs in KEP on voluntary basis. The comparative analyses by Biro *et al.*^31^^,^^33^ provide important insights into the relative performance of different KEP structures in Europe. These findings offer valuable lessons for countries with smaller or less mature KEPs, identifying areas for potential improvement and evidence-based strategies to enhance the effectiveness of their programs.

Thirdly, the development of international KEP collaborations presents a promising avenue for further expanding donor pools and optimizing match opportunities on an international scale. By transcending national borders, these programs could reduce waiting times for transplantation and improve immunological compatibility by increasing the pool of available donors. The logistical challenges, for instance prolonged CIT and high-risk donor-recipient combinations, could in the future be addressed through the integration of machine perfusion technologies such as HMP and HMPO_2_. These technologies have shown potential in preserving graft quality during transport and mitigating ischemia-reperfusion injury, particularly in the context of international exchanges.^88^

Despite the expansion and increasing sophistication of KEPs, LDKT volumes have not increased proportionally in many jurisdictions, highlighting that KEPs primarily improve matching efficiency rather than automatically expanding the underlying donor supply. Policy analyses summarizing national trends emphasize that, over the last decade, growth in DDKT has substantially outpaced growth in living donation, with living donor transplants increasing only modestly from 2014 to 2023.^16^^,^^110^

At the same time, exchange-based transplantation has become an increasingly prominent pathway within living donation; paired donations rose markedly in the US over time, and donor exchanges accounted for 20.6% of living kidney donations in 2023.^111^^,^^112^ This pattern suggests that KEP expansion often changes the composition of living donation (e.g., shifting from directed related donation towards paired/exchange pathways) without necessarily generating large net increases in the total number of living donors.

The exact causes are unknown; perhaps KEP in all its forms has mitigated a possible significant decline in LDKT; however, the expectation was that the overall number would have increased.^62^ Several upstream constraints can be identified that may limit living donor growth despite KEP expansion, including persistent financial and employment disincentives, donor medical eligibility constraints, evaluation and surgical capacity limits, and sociodemographic inequities in access to living donation.^110^ European registry analyses similarly show that increases in kidney transplantation rates have been driven more by deceased donation than living donation, and that living donor rates vary widely between countries, with higher-performing settings often combining paired exchange with donor-support measures, such as home-based education and financial compensation.^16^ For European programs, these findings underscore that international collaboration and algorithmic optimization are necessary but not sufficient. Sustained increases in living donation are likely to require coordinated policy actions that reduce donor disincentives and strengthen donor recruitment and support pathways alongside KEP development.

Although this review underscores the important role of KEPs in improving access, equity, and match quality in LDKT, several areas warrant further investigation and development.

First, the integration of advanced computational methods, including artificial intelligence and machine learning, represents a promising but largely unproven avenue. These approaches may facilitate the handling of increasingly complex donor-recipient data, support multiobjective optimization, and enhance predictive modelling of compatibility and outcomes. However, future research should focus on demonstrating their added value beyond established optimization algorithms, as well as addressing risks related to transparency, bias, and clinical interpretability.

Second, ethical, logistical, and regulatory challenges associated with international kidney exchange require careful and systematic evaluation. Further research and policy development are needed to establish equitable, transparent, and interoperable frameworks that accommodate differences in legal systems, allocation principles, reimbursement structures, and societal attitudes toward living donation, while safeguarding donor and recipient welfare.

Third, experience from mature programs suggests that expansion of KEPs alone may not be sufficient to increase population-level LDKT rates. Future strategies should therefore consider kidney exchange as part of a broader ecosystem of living donation, combining exchange programs with targeted donor recruitment, mitigation of financial and logistical disincentives, and harmonization of national and international infrastructures. In this context, the integration of national KEPs into larger, collaborative networks may maximize the clinical benefits of living donation, particularly for difficult-to-match patients, while avoiding stagnation in overall transplant activity.

### Conclusion

KEPs have evolved into a crucial component of LDKT , with the potential to significantly expand access, improve equity, and enhance long-term outcomes. By embracing innovations such as NEAD chain and voucher-based models, international collaboration, and machine perfusion technologies, KEPs can address persistent barriers and maximize their impact. Continued efforts to integrate programs, enlarge donor pools, and adopt data-driven approaches will be essential to meet the growing global demand for kidney transplantation.

## Disclosure

All the authors declared no competing interests.
